# A review on the electric vehicle routing problems: Variants and algorithms

**DOI:** 10.1007/s42524-021-0157-1

**Published:** 2021-05-19

**Authors:** Hu Qin, Xinxin Su, Teng Ren, Zhixing Luo

**Affiliations:** 1 School of Management, Huazhong University of Science and Technology, Wuhan, 430074 China; 2 Department of Management Sciences, City University of Hong Kong, Hong Kong, China; 3 School of Transportation and Logistics, Central South University of Forestry and Technology, Changsha, 410004 China; 4 School of Management and Engineering, Nanjing University, Nanjing, 210093 China

**Keywords:** electric vehicles, routing, recharging stations, exact algorithms, metaheuristics

## Abstract

Over the past decade, electric vehicles (EVs) have been considered in a growing number of models and methods for vehicle routing problems (VRPs). This study presents a comprehensive survey of EV routing problems and their many variants. We only consider the problems in which each vehicle may visit multiple vertices and be recharged during the trip. The related literature can be roughly divided into nine classes: Electric traveling salesman problem, green VRP, electric VRP, mixed electric VRP, electric location routing problem, hybrid electric VRP, electric dial-a-ride problem, electric two-echelon VRP, and electric pickup and delivery problem. For each of these nine classes, we focus on reviewing the settings of problem variants and the algorithms used to obtain their solutions.

This work was partially supported by the National Natural Science Foundation of China (Grant Nos. 71971090, 71571077, and 71531009).

## 1 Introduction

Burning fossil fuels generates greenhouse gas (GHG), which is a major cause of climate change and global warming, and also results in air pollution that damages human health (Schiffer and Walther, 2017). Transportation plays an important role in the development of economies and consumes a large portion of fossil fuels. As reported by the Environmental Protection Agency (2018), transportation activities accounted for 28.5% of GHG emissions in the US in 2016. According to Eurostat, the statistical office of the European Union (EU), fuel combustion for transportation was responsible for 25% of EU-28 GHG emissions in 2017, which has increased its contribution significantly since 1990. Governments of many countries have adopted new environmental measures and regulations to reduce GHG emissions and cut down on the consumption of fossil fuels (Keskin and Çatay, 2018).

During the last decade, electric vehicles (EVs) have received increasing attention, and their market share has increased rapidly mainly due to their environmental advantages, such as absence of GHG emission, low noise pollution, high energy efficiency, and low fuel cost (Desaulniers et al., 2016; Murakami, 2017; 2018). Moreover, EVs have two benefits for electric grids. On one hand, as most EVs are recharged at night, they can balance the excess supply of energy. On the other hand, in a smart grid, EVs can be used as a power source during peak hours in the daytime (Kempton and Letendre, 1997). Some leading express companies in the world are using EVs to serve their customers. On November 20, 2018, FedEx announced that it was expanding its fleet to add 1000 EVs that would be operated for commercial and residential pick-up and delivery services. In 2019, DHL introduced a new fleet of 63 electric cargo delivery vans to the US, with the first 30 vans going to the San Francisco Bay Area. In 2017, the chief executive of JD.com announced that the company would change its fleet of delivery vans in Beijing to EVs by the end of February 2018. China has the world's largest EV market where nearly 580000 EVs were sold in

2017, which accounts for half of the global EV market (International Energy Agency, 2018).

At present, the prevalence of the EVs still faces several challenges (Schneider et al., 2014; 2018; Schiffer and Walther, 2017). The first challenge is the limited EV driving range whose average value is approximately 200 km so far. The maximum travel range of an EV is usually less than that of a comparable gasoline-powered vehicle. Moreover, the driving range can be decreased significantly by cold temperatures. Therefore, EV drivers must be cautious when scheduling routes to ensure that EVs will not run out of electricity. The second challenge is the long charging time of EVs; fully charging the battery can take up to 8 h and the charging time increases with the battery age. The third challenge is the high cost of the EVs, especially the exorbitant battery price. The last challenge is related to the immaturity of the charging infrastructure networks, which makes recharging EVs inconvenient.

Since its introduction by Dantzig and Ramser (1959), the vehicle routing problem (VRP) and its variants have been extensively studied during the last several decades and numerous research papers can be found in the literature. The basic VRP aims to minimize the total transportation cost of visiting a set of customers by means of multiple vehicle routes starting and ending at the depot while respecting various types of constraints. For a comprehensive overview of the VRPs, we refer the reader to Toth and Vigo (2002), Golden et al. (2008), Eksioglu et al. (2009), and Braekers et al. (2016). According to the numbers of existing articles in the literature, the most representative VRP variants include the VRP with time windows (Solomon, 1987; Bräysy and Gendreau, 2005a; 2005b), location routing problem (Nagy and Salhi, 2007; Prodhon and Prins, 2014; Schneider and Drexl, 2017), dial-a-ride problem (Cordeau and Laporte, 2007; Liu et al., 2015; Ho et al., 2018; Luo et al., 2019), two-echelon capacitated VRP (Perboli et al., 2011; Baldacci et al., 2013; Liu et al., 2018), pickup and delivery problem (Savelsbergh and Sol, 1995; Nagy et al., 2015), arc routing problem (Dror, 2000; Corberán and Prins, 2010; Yu et al., 2019a), periodic vehicle routing problem (Zhang et al., 2013; Campbell and Wilson, 2014; Luo et al., 2015), and split delivery VRP (Desaulniers, 2010; Archetti and Speranza, 2012; Luo et al., 2017).

Research on the electric VRP (EVRP) was formally started with the study of the green VRP (GVRP) introduced by Erdoğan and Miller-Hooks (2012). After carefully analyzing the literature, we find that almost all of them are extensions of their corresponding VRPs. For example, the basic EVRP is a natural extension of the basic VRP and the electric location routing problem can be regarded as an extension of the location routing problem. Compared with the VRPs (see Fig. 1), the difficulty of using EVRPs results from the short driving range of the EVs and the necessity of recharging activities along the vehicle routes (see Fig. 2). Actually, the EVRPs extend the VRPs by mainly considering the following four aspects: 1) locations of the recharging (or battery swapping) stations (Yang and Sun, 2015; Schiffer and Walther, 2017; Zhang et al., 2019), 2) adoption of full or partial recharging policy (Keskin and Çatay, 2016; Sweda et al., 2017a; 2017b; Macrina et al., 2019a; 2019b), 3) charging functions (Montoya et al., 2017; Froger et al., 2019; Zuo et al., 2019), and 4) multiple types of recharging stations (Felipe et al., 2014).

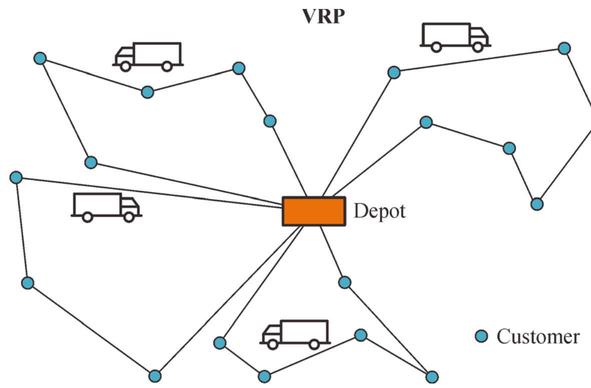

**Fig. 1** An example solution of the VRP.

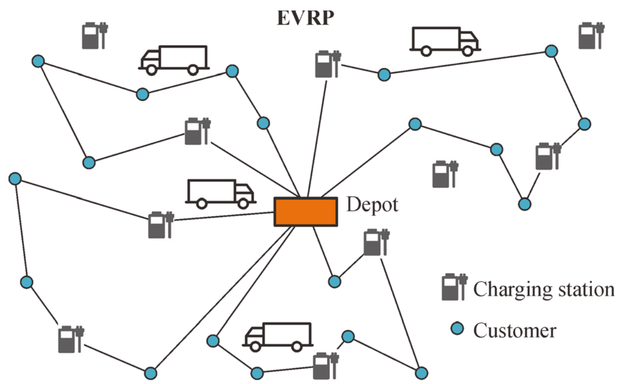

**Fig. 2** An example solution for EVRP.

In this study, we only review the routing problems that use EVs and consider recharging operations in the routes. In several existing studies, such as Doppstadt et al. (2016), Nejad et al. (2017), Sassi and Oulamara (2017), and Murakami (2017), the problems of routing EVs are investigated, but recharging EVs along the routes are not considered, and therefore we do not discuss them. Afroditi et al. (2014) surveyed the EVRPs with industry constraints, but this study includes only a small number of reference papers. Recently, Erdelić and Carić (2019) reviewed the variants and solution approaches for EVRPs. The solution approaches can be roughly divided into two main streams, namely, metaheuristics and exact algorithms. However, the authors only mentioned metaheuristics for the relevant problems. According to our statistics, the number of papers on the EVRPs published in the last two years accounts for over 40% of the total number. In Erdelić and Carić (2019), only a small number of papers in the last two years were reviewed and several problem variants were not discussed. As the research on EVRPs is in an increasing stage and the relevant literature is not rich so far, the number of articles reviewed in our work is only around 50. Figures 3 and 4 show the numbers of papers published in the recent nine years and in each relevant journal, respectively. Figure 5 displays a word cloud diagram indicating the distribution of the papers in the relative journals.

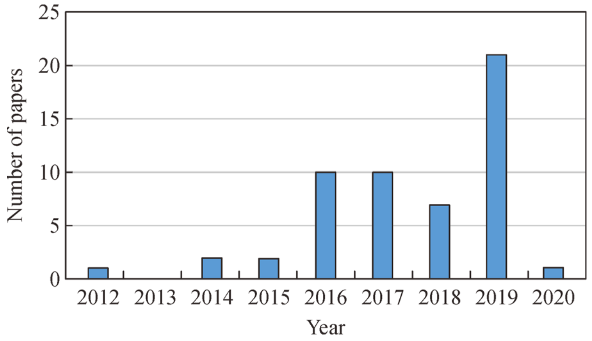

**Fig. 3** The number of papers published in recent nine years.

The rest of this paper is structured as follows. In Section 2, we review the electric traveling salesman problem (ETSP) in which only one EV is used. In Section 3, we present a thorough survey of the GVRP, which is a widely studied special case of the EVRP. In Section 4, we focus on the papers about the basic EVRP as well as its extensions that consider additional features, such as charging functions and time windows. In many situations, the carriers need to simultaneously schedule various types of vehicles, which may include EVs, hybrid EVs, and conventional vehicles. Many studies have investigated the approaches for scheduling a mixed fleet of vehicles, and thus the relevant survey on the mixed EVRP (MEVRP) is given in Section 5. Subsequently, we discuss in Section 6 all papers on the electric location routing problem (ELRP) that make decisions on both the locations of recharging facilities and the routes of EVs. In Section 7, we provide an overview of all the other EVRP variants, including hybrid EVRP (HEVRP), electric dial-a-ride problem (EDARP), electric two-echelon VRP (E2EVRP), and electric pickup and delivery problem with time windows (EPDPTW). The classes of problems surveyed in this study are presented in Fig. 6. Several variants exist in each class, and their connections and characteristics are presented in the relevant sections. Finally, Section 8 concludes the paper.

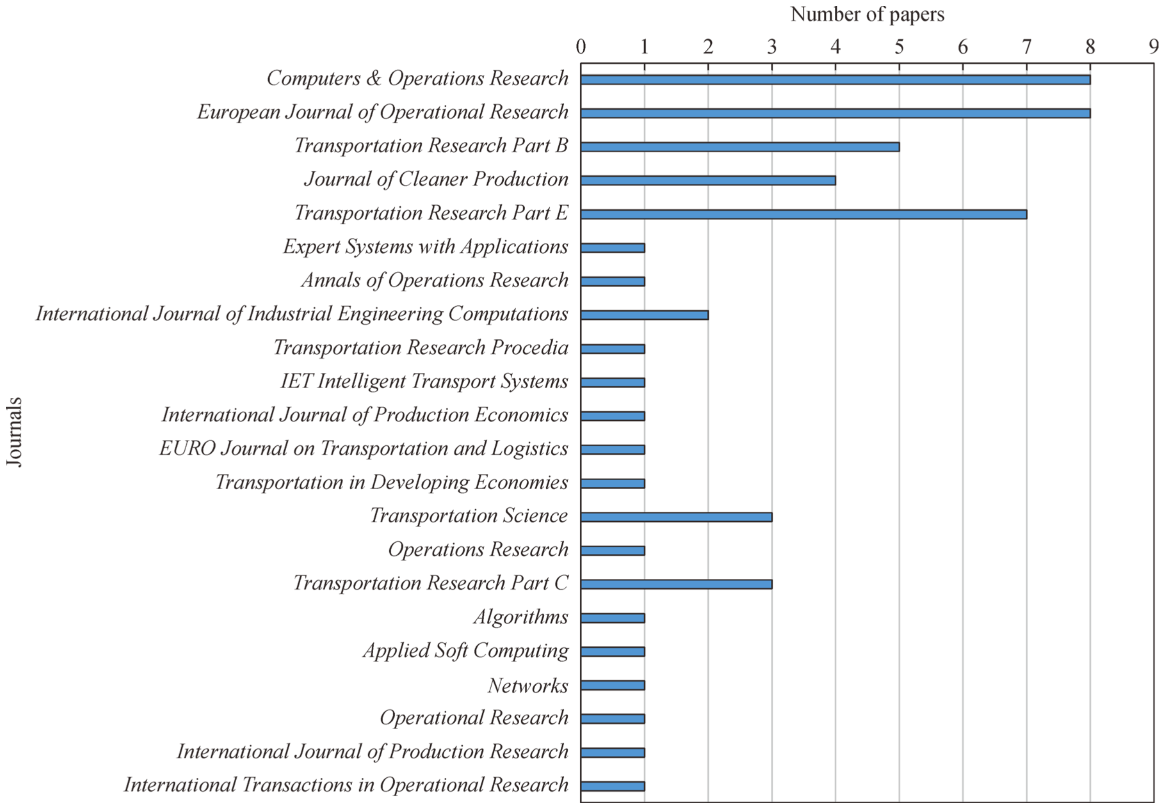

**Fig. 4** The number of papers published in each journal.

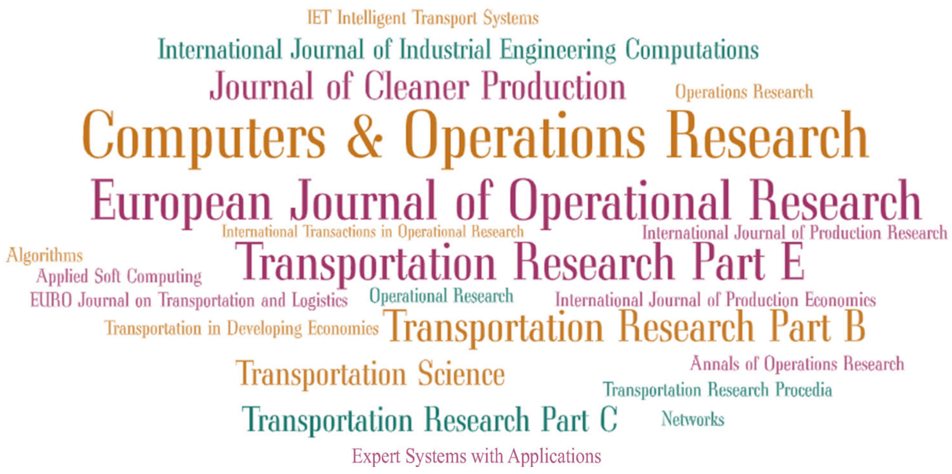

**Fig. 5** Word cloud diagram of journal names.

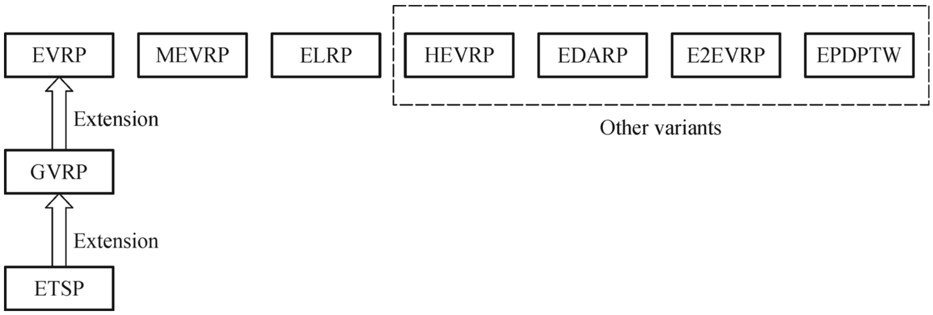

**Fig. 6** EVRP classes surveyed in this study.

## 2 Electric traveling salesman problem

Traveling salesman problem (TSP) is one of the most intensively studied problems in computational mathematics (Gutin and Punnen, 2007). The ETSP, which extends the TSP by considering only one EV, is a special case of the EVRP. When a time window is imposed on each customer, we can obtain a variant called ETSP with time windows (ETSPTW). This problem aims to find the shortest Hamiltonian tour of a set of customers while ensuring that the time window constraints are not violated and the battery level is always positive (Roberti and Wen, 2016). During the tour, the electric supply for the vehicle can be recharged in recharging stations. Roberti and Wen (2016) considered in the ETSPTW two recharging policies, namely, full recharging (the battery is fully recharged at each station) and partial recharging (any amount of electricity can be recharged at each recharging station), and presented mixed integer programming (MIP) models for the problem under both recharging policies. Subsequently, they developed a three-phase heuristic to solve the ETSPTW, where the approach components need to be simply adapted to deal with different recharging policies. This heuristic is based on the heuristics proposed by da Silva and Urrutia (2010) and Mladenović et al. (2012) for the TSP with time windows (TSPTW). At the beginning, this proposed algorithm generates a sequence of random Hamiltonian tours that do not guarantee time windows and battery capacity constraints. The three-phase heuristic is applied to each of these generated tours. First, a variable neighborhood descent (VND) procedure is used to satisfy the time window of each customer. Second, a local search heuristic based on the VND tries to reduce the cost of the route while guaranteeing the feasibility of the time window constraints. Finally, the algorithm makes the tour to satisfy the battery capacity constraints by executing a dynamic programming algorithm, which inserts recharging stations when necessary. The authors generated two sets of test instances based on the TSPTW benchmark instances available in the literature. Their approach is able to find optimal solutions for most of the instances with 20 customers under both recharging policies. They also adapted their approach to deal with the TSPTW instances and obtained similar results, compared with the general variable neighborhood search (VNS) of da Silva and Urrutia (2010).

In the market, various types of recharging stations exist, which use different charging technologies. As a result, the charging rate at one type of recharging station may differ from that of another. Küçükoğlu et al. (2019) extended the ETSPTW by considering various charging rates and introduced a new problem called ETSPTW with mixed charging rates (ETSPTW–MCR). To solve this problem, they proposed a new and effective hybrid simulated annealing/tabu search (SA/TS) algorithm. This algorithm improved on the existing hybridization of SA and TS by employing an efficient search strategy, a modified solution acceptance criterion, and two types of tabu lists. The ETSPTW–MCR is solved by a two-state procedure. First, the hybrid SA/TS algorithm seeks the least cost solutions only considering the constraints in the TSPTW. Then, a dynamic programming algorithm is executed to obtain feasible solutions for the ETSPTW–MCR. For a given route that only contains customers, the dynamic programming algorithm generates recharging operation plans optimally by inserting the recharging stations in the customer-only route. In the experiments, the authors employed their algorithm to solve the instances of the TSPTW, ETSPTW, and ETSPTW–MCR. Compared with the existing algorithms for the TSPTW and ETSPTW, their algorithm achieved competitive results. In addition, the results generated by their algorithm is stable because the standard deviations of most of the results are zero or very close to zero. The ETSP and its variants are depicted in Fig. 7, where for each arc the problem at its end is an extension of the problem at its origin.

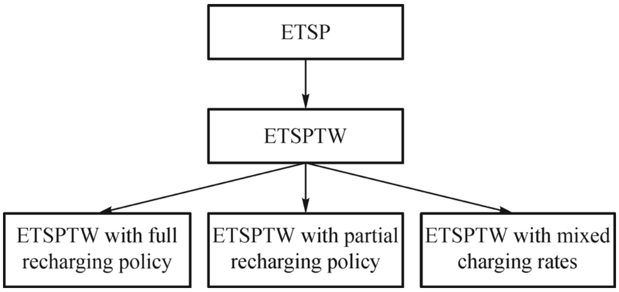

**Fig. 7** The ETSP and its variants.

## 3 Green vehicle routing problem

The GVRP, formally introduced by Erdoğan and Miller-Hooks (2012), is a special case of the EVRP that does not consider the vehicle capacity. This problem is defined on a directed graph $G=(V, E)$, where the vertex set $V=N\cup F\cup\{0\}$ consists of a set $N=\{1, ..., n\}$ of $n$ customers, a set $F=\{n+1, ..., n+f\}$ of $f$ recharging stations, and vertex 0 representing the depot. Each customer $i\in N$ has a service time $S_i$ and each edge is associated with a distance $d_{i,j}$ and a travel time $t_{i,j}$. These $n$ customers need to be served by an unlimited number of homogeneous alternative fuel vehicles (AFVs) positioned at the depot. Each AFV has a maximum driving time of $T$ minutes and a fuel capacity $Q$. The vehicle fuel consumption is assumed to be linearly proportional to the distance traveled. If $h$ is the constant rate of fuel consumption per unit distance, the maximum distance $D$ that a vehicle can travel without refueling is computed as $D=Q/h$. Each AFV can be refueled at any of the refueling stations. It is assumed that the AFVs must be fully refueled and each stop at the refueling station incurs a constant operation time of $S$ minutes. The GVRP seeks to find a set of at most $m$ tours that start from and end at the depot and serve all customers with the aim of minimizing the total travel distance. Information on the solution approaches for the GVRP is summarized in Table 1.

Erdoğan and Miller-Hooks (2012) proposed an MIP model for the GVRP and developed two constructive heuristics to solve it. The first constructive heuristic was modified from the Clarke–Wright saving algorithm (Clarke and Wright, 1964), which was designed for the classical VRP. The second one is the density-based clustering algorithm, which was built on the concept from the density-based spatial clustering of applications with noise algorithm (Ester et al., 1996). The routes generated by the two heuristics are further improved through two techniques that apply, respectively, an inter- and intra-route edge exchange operator. The authors tested their approaches based on a set of 40 self-generated instances with 20 customers and 12 real cases with up to 500 customers. The experimental results show that their heuristics performed well. Koç and Karaoglan (2016) proposed a new MIP model for the GVRP and implemented a branch-and-cut algorithm to solve this problem. During the process of the branch-and-cut algorithm, an SA algorithm that involves four neighborhood search operators is employed to obtain feasible solutions for the problem. In their experiments, Koç and Karaoglan (2016) compared their proposed model with that from Erdoğan and Miller-Hooks (2012); the results show that the new model can generate better lower bounds. The branch-and-cut algorithm optimally solved 22 out of 40 small instances that contain 20 customers. Montoya et al. (2016) proposed an effective two-phase heuristic to tackle the GVRP. In the first stage, this heuristic builds a pool of routes via a set of randomized route-first cluster-second heuristics (Beasley, 1983; Prins et al., 2014) with an optimal procedure of inserting alternative fuel stations. In the second phase, the heuristic seeks feasible GVRP solutions by solving a set-partitioning formulation based on the routes in the pool. The authors conducted experiments using 52 instances in Erdoğan and Miller-Hooks (2012), and then compared their results to the best results obtained by Erdoğan and Miller-Hooks (2012), Schneider et al. (2014; 2015), and Felipe et al. (2014). Numerical results reveal that this heuristic yielded new best-known solutions for 8 instances, achieved the best solutions for 40 instances, and generated

**Table 1** Solution approaches for GVRP

| References | Approaches | Results | Advantages | Disadvantages |
|---|---|---|---|---|
| Erdoğan and Miller-Hooks (2012) | Constructive heuristics | Tested on a set of 40 self-generated instances with 20 customers and 12 real cases with up to 500 customers and performed well | Fast | Low quality |
| Koç and Karaoglan (2016) | Branch-and-cut algorithm | Optimally solved 22 out of 40 small instances with 20 customers | Optimal solution | Slow |
| Montoya et al. (2016) | Two-phase heuristic | Yielded new best-known solutions for 8 instances, and achieved the best solutions for 40 instances | Fast | Near-optimal solution |
| Yavuz (2017) | Iterated beam search algorithm | Outperformed CPLEX and all previous heuristics on large instances | Be able to either exactly or heuristically solve the problem | – |
| Leggieri and Haouari (2017) | MIP model-based reduction procedure | Better than the branch-and-cut algorithm proposed by Koç and Karaoglan (2016) | Optimal solution | Slow |
| Andelmin and Bartolini (2017) | Set-partitioning model based exact algorithm | Optimally solved instances with up to 110 customers | Optimal solution | Slow |
| Affi et al. (2018) | Variable neighborhood search heuristic | Produced the best solution values for 11 out of 12 large instances and was superior to the other existing heuristics in the literature | Fast | Near-optimal solution |
| Andelmin and Bartolini (2019) | Multi-start local search heuristic | Found 8 new best-known solutions and reached the best-known solution for the rest of the instances | Fast | Near-optimal solution |

solutions that are very close to the best-known solutions for the 4 remaining instances.

Yavuz (2017) developed an iterated beam search (IBS) algorithm to heuristically or exactly solve the GVRP. This IBS algorithm constructs a search tree and is able to explore the solution space either partially or completely. The search tree consists of nodes and branches, where the leaf nodes correspond to the complete solutions and the other nodes represent partial solutions. This algorithm can employ different lower- and upper-bound strategies, and can be accelerated by a dominance strategy. Yavuz (2017) tested their algorithm on instances provided by Erdoğan and Miller-Hooks (2012) and Yavuz and Çapar (2017). The experimental results show that the exact IBS algorithm outperforms CPLEX 12.6 on a set of 30 instances obtained from Yavuz and Çapar (2017). Moreover, for the larger instances, this algorithm also works better, compared with the best-known solutions in the literature. Leggieri and Haouari (2017) designed a practical solution approach that uses a novel MIP model and a reduction procedure to solve the GVRP. The new model has two advantages. First, it includes a polynomial number of variables and constraints, and thus can be solved by commercial MIP solvers. Second, it is flexible and can be easily adapted to handle many variants of the problem. In addition, the authors presented reduction procedures for early fixing binary variables to reduce the problem size and speed up the computation. They tested their model on instances used by Erdoğan and Miller-Hooks (2012) and demonstrated that directly solving their model can yield better results than the branch-and-cut algorithm presented by Koç and Karaoglan (2016).

Andelmin and Bartolini (2017) proposed an exact algorithm to solve the GVRP, and formulated the problem into a set-partitioning model based on a multigraph in which columns correspond to feasible routes. This model is strengthened by three classes of valid inequalities, namely, weak subset row inequalities, subset row inequalities, and $k$-path cuts. The proposed exact method follows the general schema presented by Baldacci et al. (2008; 2011). The authors conducted experiments on two set of instances, namely, a set of instances from Erdoğan and Miller-Hooks (2012) and a set of self-generated instances. The computational results show that this algorithm can optimally solve instances with up to 110 customers. Based on the multigraph reformulation introduced by Andelmin and Bartolini (2017), Andelmin and Bartolini (2019) also devised a multi-start local search heuristic for the GVRP. This heuristic consists of three phases: The first two phases iteratively construct new solutions, improve them by a local search procedure, and then store all vehicle routes encountered in a route pool; and the last phase optimally combines vehicle routes in the route pool by solving a set-partitioning model and improves the resultant solution by a local search procedure. The authors tested their heuristic using 52 instances from Erdoğan and Miller-Hooks (2012) and 40 instances generated by themselves. For the set of the former 52 instances, this heuristic found 8 new best-known solutions and reached the best-known solutions for the rest of the instances. For the set of the 40 instances, it found 23 optimal solutions and achieved upper bounds whose average value is only within 0.27% far from the optimal solution value. Affi et al. (2018) tackled the GVRP using a VNS heuristic (Mladenović and Hansen, 1997) and also tested their algorithm on 52 GVRP instances presented by Erdoğan and Miller-Hooks (2012). When compared with the results in the literature, this VNS algorithm provided the best solution values for all small instances with less computation time. For the larger instances, it also produced the best solution values for 11 out of 12 instances, and its performance is superior to the other existing heuristics in the literature.

## 4 Electric vehicle routing problem

The EVRP is a straightforward extension of the classic VRP by involving EVs and the operations of recharge. This section first provides an MIP model for the standard EVRP and then introduces its several types of extensions and variants. In the literature, the EVRP has been extended by considering many features, such as multiple types of recharging stations, minimization of the total energy consumed, multiple depots, energy consumption uncertainty, heterogeneous EVs, time windows, and nonlinear charging functions. According to the number of articles published, we find the two main extensions of the EVRP are that with time windows (Schneider et al., 2014) and that with nonlinear charging functions (Montoya et al., 2017). The EVRP and its variants are summarized in Table 2, which shows the problem names, features considered, and solution approaches.

Here, we present the MIP model of the standard EVRP. Let $V'$ be the set of vertices with $V' = V \cup F'$, where $V = \{1, ..., n\}$ denotes the set of $n$ customers and $F'$ is the set of dummy vertices related to the set $F$ of recharging stations. Vertices 0 and $n+1$ represent the exit and entrance of the depot, and each route must start from vertex 0 and end at vertex $n+1$. Moreover, we define $F'_0 = F' \cup \{0\}$, $V'_0 = V' \cup \{0\}$, and $V'_{0,n+1} = V' \cup \{0, n+1\}$. The EVRP is defined on a complete and directed graph $G = (V'_{0,n+1}, E)$ with the set of edges $E = \{(i, j) | i, j \in V'_{0,n+1}, i \neq j\}$. Each edge has a distance $d_{i,j}$, a travel time $t_{i,j}$, and a constant battery consumption rate $h$ (per unit distance), i.e., traversing this edge consumes $hd_{i,j}$ battery charge. A fleet of identical EVs with a loading capacity of $C$ and a battery capacity of $Q$ is positioned at the depot. When leaving the depot, EVs have full battery power. Each vertex $i \in V'_{0,n+1}$ has a positive demand $q_i$, which is 0 if $i \notin V$, and a service time $s_i$ ($s_0 = s_{n+1} = 0$). At each recharging station, the difference between the present battery level and $Q$ is recharged with a charging rate of $g$ (i.e., full recharging policy is adopted). Each customer must be visited by exactly one vehicle, i.e., split delivery is not allowed. We define decision variable $\tau_i$ as the time of arrival, decision variable $u_i$ as the remaining cargo, and decision variable $y_i$ as the remaining battery level on arrival at vertex $i \in V'_{0,n+1}$. Let $x_{i,j}$ ($i \in V'_0, j \in V'_{n+1}, i \neq j$) be a binary decision variable that equals 1 if edge $(i, j)$ is traversed and 0 otherwise. The objective of this problem is to minimize the total traveling distance. The MIP model for the EVRP is described as follows:

$$\min \sum_{i \in V'_0,\ j \in V'_{n+1},\ i \neq j} d_{i,j} x_{i,j} \tag{1}$$

*s.t.*

$$\sum_{j \in V'_{n+1},\ i \neq j} x_{i,j} = 1, \ \forall i \in V, \tag{2}$$

$$\sum_{j \in V'_{n+1},\ i \neq j} x_{i,j} \leqslant 1, \ \forall i \in F', \tag{3}$$

$$\sum_{j \in V'_{n+1},\ i \neq j} x_{j,i} - \sum_{i \in V'_0,\ i \neq j} x_{i,j} = 0, \ \forall j \in V', \tag{4}$$

$$\tau_i + (t_{i,j} + s_i) x_{i,j} - M(1 - x_{i,j}) \leqslant \tau_j,$$

$$\forall i \in V_0, j \in V'_{n+1}, i \neq j, \tag{5}$$

$$\tau_i + t_{i,j} x_{i,j} + g(Q - y_i) - (M + gQ)(1 - x_{i,j}) \leqslant \tau_j,$$

$$\forall i \in F', j \in V'_{n+1}, i \neq j, \tag{6}$$

$$0 \leqslant u_j \leqslant u_i - q_i x_{i,j} + C(1 - x_{i,j}),$$

$$\forall i \in V'_0, j \in V'_{n+1}, i \neq j, \tag{7}$$

$$0 \leqslant u_0 \leqslant C, \tag{8}$$

$$0 \leqslant y_j \leqslant y_i - hd_{i,j} x_{i,j} + Q(1 - x_{i,j}),$$

$$\forall j \in V'_{n+1}, i \in V, i \neq j, \tag{9}$$

$$0 \leqslant y_j \leqslant Q - hd_{i,j} x_{i,j}, \ \forall i \in F'_0, j \in V'_{n+1}, i \neq j, \tag{10}$$

$$x_{i,j} \in \{0, 1\}, \ \forall i \in V'_0, j \in V'_{n+1}, i \neq j. \tag{11}$$

In the above model, $M$ is a sufficiently large positive number. Objective (1) minimizes the total traveling distance. Constraint (2) ensures that each customer must be served exactly once, and constraint (3) states that each dummy recharging station must be visited at most once. Constraint (4) represents flow conservation constraints. Constraints (5) and (6) define the relationship of $u_i$ and $u_j$, which are associated with two consecutively visited vertices $i$ and $j$. Constraints (7) and (8) guarantee demand fulfillment for each customer. Finally, constraints (9) and (10) guarantee that the battery charge level never falls below 0.

**Table 2** EVRP and its variants

| Reference | Problem name | Features | Approaches |
|---|---|---|---|
| Zhang et al. (2018a) | Capacitated GVRP | Zero recharging time | Two-phase heuristic and metaheuristic based on ant colony system |
| Granada-Echeverri et al. (2020) | EVRP with backhaul | Two types of customers | Iterated local search heuristic |
| Felipe et al. (2014) | GVRP with multiple technologies and partial recharges | Multiple recharging technologies and partial recharging | Constructive heuristic, variable neighbor-hood search heuristic, and simulated annealing algorithm |
| Lin et al. (2016) | General EVRP | Both delivering or collecting products, and vehicle load | MIP model |
| Shao et al. (2018) | EVRP | Fixed charging time | Hybrid genetic algorithm |
| Zhang et al. (2018b) | EVRP | Full recharging policy, and zero recharging time | Ant colony algorithm and adaptive large neighborhood search heuristic |
| Li et al. (2019) | Multi-depot GVRP | $CO_2$ emissions | Improved ant colony optimization algorithm |
| Pelletier et al. (2019) | EVRP with energy consumption uncertainty | Energy consumption uncertainty | Cutting-plane algorithm and two-phase heuristic based on large neighborhood search |
| Schneider et al. (2014) | EVRPTW with recharging stations | Time windows | Hybrid heuristic that combines variable neighborhood search algorithm and tabu search heuristic |
| Desaulniers et al. (2016) | EVRPTW | Time windows | Branch-and-price-and-cut algorithm |
| Keskin and Çatay (2016) | EVRPTW | Time windows and partial recharging policy | Adaptive large neighborhood search heuristic |
| Hiermann et al. (2016) | Electric fleet size and mix VRP with time windows and recharging stations | Time windows and heterogeneous EVs | Branch-and-price algorithm and adaptive large neighborhood search heuristic |
| Zhao and Lu (2019) | Real-world EVRP | Heterogeneous EVs, full recharging policy, and constant recharging time | Heuristic based on adaptive large neighborhood search heuristic and set-partitioning model |
| Yu et al. (2019b) | Heterogeneous fleet GVRPTW | Heterogeneous EVs, full recharging policy, and carbon emissions | Branch-and-price algorithm |
| Wen et al. (2016) | EV scheduling problem | Full recharging policy, multiple depot, and variable recharging time | Adaptive large neighborhood search heuristic |
| Wang et al. (2019) | Multi-depot GVRP with shared transportation resource | Multiple depot, shared transporta-tion resources, time-dependent speed, and piecewise penalty costs for violating time windows | Hybrid heuristic |
| Keskin and Çatay (2018) | EVRP with time windows and fast chargers | Partial recharging and multiple types of rechargers | Two-phase matheuristic approach |
| Verma (2018) | EVRP with time windows, recharging stations, and battery swapping stations | Both chargers and batteries for swapping | Two-step heuristic |
| Kancharla and Ramadurai (2018) | EVRP with load-dependent energy consumption | Load-dependent energy consumption | Adaptive large neighborhood search heuristic |
| Cortés-Murcia et al. (2019) | EVRP with time windows, partial recharges, and satellite customers | Partial recharges and satellite customers | Hybrid heuristic consisting of iterated local search, variable neighborhood decent, and set-partitioning model |
| Montoya et al. (2017) | EVRP with nonlinear charging function | Nonlinear charging function | Hybrid metaheuristic that combines an iterated local search algorithm and a concentration heuristic |
| Froger et al. (2019) | EVRP with nonlinear charging function | Nonlinear charging function | Heuristic and exact labeling algorithm |
| Zuo et al. (2019) | EVRPTW with concave nonlinear charging function | Nonlinear charging function | No tailored solution procedure |

Zhang et al. (2018a) studied a capacitated GVRP, which is a special case of the EVRP without considering recharging time, and proposed two solution approaches, namely, a two-phase heuristic and a metaheuristic based on the ant colony system to solve the problem. In the first stage of the two-stage heuristic, a TSP is solved using the nearest neighborhood greedy algorithm to find a delivery tour. In the second stage, according to the remaining fuel and remaining products, recharging stations and the depot are inserted in the TSP tour to generate a feasible solution. The authors randomly generated benchmark instances, which include a set of 10 small instances with the number of customers ranging from 15 to 24 and the number of recharging stations fixed to 2; and furthermore, a set of 20 large instances consider 25, 50, 70, 100, and 150 customers and 2, 4, 6, and 8 recharging stations. The experimental results show that the performance of the ant colony system is 38.27% better than that of the two-stage heuristic. Granada-Echeverri et al. (2020) studied an EVRP that provides services for two sets of customers. The first set includes the customers that require a given quantity of products to be delivered and the second set includes the customers that need to send a given quantity of products to the depot. Additionally, this problem requires the customers in the first set to be visited before those in the second set. To solve the problem, the authors proposed an iterated local search algorithm that applies inter- and intra-route operations to explore the search space associated with the solution encoding strategy.

To the best of our knowledge, the number of existing papers that investigated the standard EVRP is extremely limited. In fact, most of the papers in the literature studied the extensions and variants of the standard EVRP. Felipe et al. (2014) introduced an EVRP with multiple technologies and partial recharging. Different from the standard EVRP, this problem considers various types of recharging stations and allows any amount of electricity to be recharged at the recharging station. Each type of recharging station has a given recharge speed and recharge unit cost. The objective of this problem is to minimize the total recharging cost, which is composed of a fixed cost and a variable cost. The fixed cost of each recharge operation is given by the cost of a battery divided by the estimated maximum number of recharge operations. The variable cost of a recharge operation is proportional to the amount of electricity recharged and also depends on the type of recharging station. The authors implemented three types of heuristics to solve their problem. The first type is a constructive heuristic that aims to generate feasible solutions using a short computation time. The second type is the VNS heuristic that employs three neighborhood search operators, namely, recharge relocation, 2-opt, and reinsertion. The last type is the SA algorithm. Lin et al. (2016) mentioned a general EVRP whose objective is to minimize the total cost, consisting of the battery charging cost, travel time cost, and battery-charging waiting time cost. In addition, this problem can also deal with the operations of both delivering and collecting products. During the process of calculating the cost, the vehicle load and travel speed are considered while the travel speed on each edge is assumed to be a known constant and the vehicle load varies along with the route. Through a case study, Lin et al. (2016) showed that the load effect cannot be ignored in the routing strategies.

Shao et al. (2018) presented an EVRP that minimizes the sum of the fixed vehicle cost, travel cost, and charging cost. This work assumed that the charging time is a constant, and a full recharging policy is adopted. To solve this problem, the researchers implemented a hybrid genetic algorithm, which is a combination of a genetic algorithm and a local search procedure. Zhang et al. (2018b) introduced an EVRP that seeks to minimize the total energy consumed by all EVs. The amount of consumed energy depends on several factors, such as traveling distance, vehicle weight, vehicle speed, and engine efficiency. In this problem, the full recharging policy is also adopted, while the time consumed by the recharging operation is not considered, i.e., the service time at each recharging station is zero. As the solution approaches to their problem, Zhang et al. (2018b) proposed an ant colony algorithm and an adaptive large neighborhood search (ALNS) heuristic (Ropke and Pisinger, 2006). The authors conducted experiments based on a set of self-generated instances and found that the ant colony algorithm is able to provide close-to-optimal solutions for the small-sized instances and performs better than the ALNS heuristic for the large-sized instances in terms of solution quality and computation time. Li et al. (2019) studied a multi-depot EVRP that has four objectives, namely, maximizing revenue, minimizing cost, minimizing the traveling time of vehicles, and minimizing the level of $CO_2$ emissions. They solved their problem by applying an improved ant colony optimization algorithm.

In practice, the amount of energy consumed is uncertain due to factors such as weather and road conditions, driver behavior, and several energy consumption parameters that are difficult to determine precisely. To deal with this issue, Pelletier et al. (2019) introduced an EVRP with energy consumption uncertainty and then proposed a robust optimization framework to address the aforementioned uncertainties. Different from the deterministic EVRP, this problem assumes that each edge $(i, j)$ has an expected empty-vehicle energy consumption $a_{i,j}$ (kWh) and an expected load-dependent energy consumption rate $b_{i,j}$ (kWh/kg). The realized energy consumption value deviates from the expected values because of the uncertain environments. The authors formulated their stochastic problem to a deterministic MIP model whose objective function is to minimize the total fixed cost of EVs, the total maintenance cost proportional to the total traveling distance, and worst-case energy cost. The authors first used a cutting-plane algorithm to produce the optimal solutions for the small instances of the problem. Thereafter, they developed a two-phase heuristic approach based on the large neighborhood search (LNS) to seek near-optimal solutions for the large instances.

### 4.1 EVRP with time windows

Schneider et al. (2014) initiated the research on EVRP with time windows (EVRPTW). The MIP model of the EVRPTW can be obtained by adding time window constraints to the model (1)–(11). The authors implemented a hybrid heuristic that combines VNS algorithm and TS heuristic (henceforth called VNS/TS for brevity) to solve the EVRPTW. They conducted numerical experiments to evaluate the performance of their method. First, they compared the performance of their VNS/TS heuristic and CPLEX using small-sized instances. The results show that the VNS/TS heuristic is able to solve these small instances to optimality within only a few seconds. However, for most of these instances, CPLEX consumed much more computation times. Moreover, for 11 out of 36 small instances, CPLEX cannot produce provable optimal solutions. Second, the authors analyzed the efficiency of the algorithmic components of their hybrid heuristic based on a set of medium-size instances. Finally, they demonstrated the ability of the VNS/TS heuristic on solving the instances of the GVRP, VRPTW, and multi-depot VRP with inter-depot routes (Crevier et al., 2007). Desaulniers et al. (2016) designed exact branch-and-price-and-cut algorithms for four versions of the EVRPTW, which are the following: 1) each route is allowed to visit at most one recharging station and a full recharging policy is adopted, 2) each route can visit multiple recharging stations and a full recharging policy is adopted, 3) each route is allowed to visit at most one recharging station and a partial recharging policy is adopted, and 4) each route can visit multiple recharging stations and a partial recharging policy is adopted. Actually, the second version is exactly the same as the problem studied by Schneider et al. (2014). The branch-and-price-and-cut algorithm follows a standard framework including column generation procedure, cutting planes, and branching strategies. Keskin and Çatay (2016) focused on the EVRPTW with the partial recharging policy and developed an ALNS heuristic to solve it efficiently. This ALNS heuristic uses new removal and insertion mechanisms to handle the structure of the problem, which includes customer removal, recharging station removal, customer insertion, and recharging station insertion. The authors applied their heuristic to the instances of the EVRPTW with the full recharging policy and achieved new best-known solutions for four instances. Further, they showed that the partial recharging policy may improve the solutions substantially, compared with the full recharging one.

Other works in the literature focused on the extensions of the EVRPTW. Hiermann et al. (2016) extended the EVRPTW by considering heterogeneous EVs, and the resultant problem is called the heterogeneous EVRPTW (HEVRPTW). The available vehicle types differ in their load capacities, battery capacities, amount of energy consumed per distance unit, recharging time per energy unit, and acquisition costs. The authors provided two models for the HEVRPTW, namely, an arc-flow model and a set-partitioning model. Then, they applied the branch-and-price algorithm to solve the set-partitioning model for the optimal solution of the problem. However, the instance size that can be optimally solved by this exact method is limited. To handle the instances of real sizes, they also developed a heuristic based on the ALNS heuristic. In this heuristic, a local search procedure is used to intensify the search process in each iteration. As the ALNS heuristic has low ability to better position the recharging stations, a post-processing procedure is added to improve the selection and positioning of recharging stations for a route with the fixed customer visiting order. This post-optimization procedure is realized by a labeling algorithm and assumes that at most one recharging station can be placed between any two consecutive customers to reduce the computation time. Zhao and Lu (2019) also studied an HEVRPTW in which a full recharging policy is adopted, and the charging time of each EV type is assumed to be constant regardless of the remaining battery power of the EV. The objective of this problem is to minimize the sum of the EV acquisition, travel, charging, and waiting costs incurred by the customers and distribution center. The researchers also devised a heuristic approach based on the ALNS heuristic and the set-partitioning model to solve their problem. This heuristic first applies the ALNS heuristic to search the solution space and stores the feasible routes encountered during the search process in a route pool. Then, it constructs a set-partitioning model using the routes in the pool and solves it using an MIP solver. As the route pool is a subset of all feasible routes, the optimal solution of the set-partitioning model can only be regarded as a near-optimal solution of the problem. Yu et al. (2019b) studied a GVRP with time windows (GVRPTW) that employs heterogeneous EVs and tries to minimize the total carbon emissions. This problem considers the vehicle capacity, and thus it is essentially the HEVRPTW. The authors developed a branch-and-price algorithm to solve their problem to optimality.

Wen et al. (2016) introduced a multi-depot EVRPTW that employs the full recharging policy and considers recharging time proportional to the charged electricity. Each vertex to be served has a fixed starting time, which is equivalent to having a service time window whose starting and ending times are the same. Moreover, each depot or recharging station has a specified time window within which the EV can visit. The authors implemented an ALNS heuristic to find near-optimal solutions for their problem. Wang et al. (2019) extended the multi-depot EVRPTW by considering shared transportation resources, where the time-dependent speed and piecewise penalty costs for violating the time windows are also incorporated. The researchers proposed a bi-objective model to minimize the total carbon emission and operational cost, and then designed a hybrid heuristic to solve the problem. Keskin and Çatay (2018) extended the EVRPTW by allowing partial recharging and considering three types of chargers equipped at each recharging station, namely, normal, fast, and super-fast chargers. The faster charger uses less time while the unit cost of energy is higher. This problem aims to first minimize the number of vehicles and then minimize the total cost of energy consumed. To solve this problem, the authors proposed a two-phase matheuristic approach, where the ALNS heuristic is applied to find near-optimal solutions in the first phase and an exact method with the aid of the MIP solver is used in the second phase to further improve the solution quality. Specifically, the exact approach uses a simplified MIP model that fixes the sequence of the customers, and makes decisions based on the selection of the recharging stations and charger types, and the amount of energy charged.

Battery swapping is an alternative way to recharge EVs. Usually, the battery of the EV can be swapped with a new fully recharged one within only five minutes. Verma (2018) introduced a variant of the EVRPTW in which the recharging stations provide both chargers and batteries for swapping. To solve this problem, they designed a heuristic that consists of two steps. In the first step, the number of visits to each recharging station is fixed using a local search procedure that is combined with efficient lower and upper bounds. In the second step, this heuristic determines the routing costs of the delivery plan using a genetic algorithm. In the heuristic, the constraints on battery capacity and time windows can be violated. Vehicle load can affect the energy consumption as well as the transportation cost, which has been mentioned in several studies (Zhang et al., 2012; Luo et al., 2017). Kancharla and Ramadurai (2018) extended the EVRPTW in which energy consumption depends on the vehicle load. Actually, the electricity consumption per distance unit of an EV is a function of its speed and load. The authors applied an ALNS heuristic with several special operators to seek the near-optimal solution of their problem. Cortés-Murcia et al. (2019) introduced a new variant of the EVRPTW that involves the concept of satellite customers. During the time for recharging the EV at any recharging station, if the cargoes on the EV can be delivered to a certain customer by alternative transportation modes, such as walking, bicycles, and drones, such customers are called satellite customers. It is assumed that during each recharging operation, at most one satellite customer can be visited. This extended problem is solved by a hybrid heuristic consisting of iterated local search, variable neighborhood descent, and set-partitioning model.

### 4.2 EVRP with nonlinear charging function

The majority of the existing studies on routing EVs assume that the recharging time is a linear function of the amount of electricity charged. However, the charging duration can be roughly divided into two stages, where in the first stage charging time and charged electricity have a linear relationship and in the second stage its relationship is nonlinear. To face this reality, Montoya et al. (2017) incorporated into the EVRP model the nonlinear feature of charging process, and thus the resultant problem is called the EVRP with nonlinear charging function (EVRP–NL). As shown in Fig. 8, the charging process can be approximated to a piecewise linear function. The authors provided an MIP model for the EVRP–NL and then developed a hybrid metaheuristic that combines an iterated local search algorithm and a concentration heuristic to solve it. Compared with the model used by Montoya et al. (2017), two new MIP models were proposed by Froger et al. (2019) for the EVRP–NL. Subsequently, they proposed a heuristic and an exact labeling algorithm to solve this problem. Extensive experiments were conducted to compare the results of directly solving the MIP models using Gurobi 7.5.0, the heuristic and exact labeling algorithm. Zuo et al. (2019) focused on formulating the EVRP–NL into new MIP models that do not use duplicated dummy vertices or edges. In this work, no tailored solution procedure is given. Through experiments, they compared the performance of their models with that of the traditional models, and demonstrated the superiority of their models.

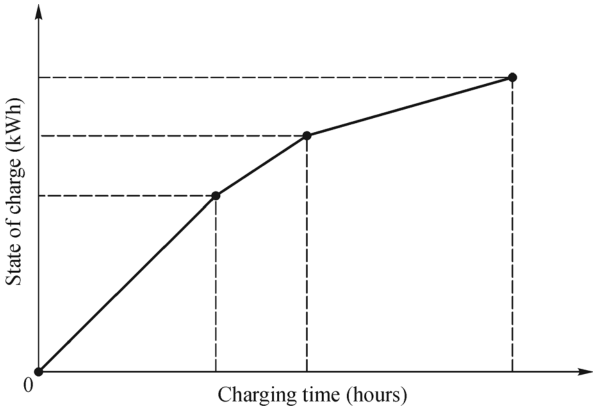

**Fig. 8** Piecewise linear approximation of nonlinear charging function.

## 5 Mixed electric vehicle routing problem

Many companies own both EVs and conventional vehicles (CVs). Therefore, routing a mixed fleet of EVs and CVs becomes necessary in many situations. The energy costs of EVs are lower, whereas labor costs may increase due to time spent on the recharging operations. Several works have investigated the problems of scheduling a mixed fleet of EVs and CVs, which are presented in Table 3. Goeke and Schneider (2015) proposed an EVRP that considers time windows and a mixed fleet of EVs and CVs. The energy consumption functions of these vehicles are related to the vehicle speed, gradient, and vehicle load. The authors formulated their problem into a nonlinear MIP

**Table 3** MEVRP and its variants

| Reference | Problem name | Features | Approaches |
|---|---|---|---|
| Goeke and Schneider (2015) | EVRP with time windows and mixed fleet | Energy consumption functions | Adaptive large neighborhood search heuristic |
| Hiermann et al. (2019) | Hybrid heterogeneous electric fleet routing problem with time windows and recharging stations | Time windows, conventional, plug-in hybrid, and electric vehicles | Metaheuristic consisting of a genetic algorithm, a local search procedure and a large neighborhood search procedure |
| Macrina et al. (2019a) | Green mixed fleet VRP with partial battery recharging and time windows | Partial battery recharging and time windows | Iterated local search heuristic |
| Macrina et al. (2019b) | Energy-efficient GVRP with mixed vehicle fleet, partial battery recharging, and time windows | Partial battery recharging, time windows, energy consumption function, effects of acceleration and braking phases, and battery lifespan | Large neighborhood search heuristic |

model, which cannot be directly handled by general solvers such as CPLEX and Gurobi. To find a high-quality solution for this problem, the researchers developed an ALNS heuristic that is enhanced by a local search procedure for intensifying the search process. This ALNS heuristic has three main features. First, an adaptive mechanism is used to select the number of customers to be removed by the removal operators in each iteration. Second, it uses surrogate violations to avoid the calculation of the time window and battery capacity violations. Finally, it adopts an acceptance criterion that considers the different penalty factors used when computing the solution objective value.

Hiermann et al. (2019) studied a problem that simultaneously schedules CVs, EVs, and plug-in hybrid EVs (HEVs). The HEVs have two engines, namely, an internal combustion engine and an electric engine, and thus can avoid visiting to recharging stations by switching to the internal combustion engine. The travel cost per distance unit when using the internal combustion engine is higher than that caused by the electric engine. To solve this complex problem, Hiermann et al. (2019) designed a metaheuristic consisting of a genetic algorithm, a local search procedure, and an LNS procedure. Evaluating the quality of a given route is difficult because optimally deciding the visits to recharging stations, arrival times, and possible engine mode changes are computationally expensive. As a result, to approximately calculate the cost of each route, the authors adopted a heuristic evaluation method that requires less computation time. Specifically, the approach of evaluating routes is based on a layered optimization algorithm that combines a labeling technique and several greedy evaluation policies for inserting recharging stations and selecting the engine mode. Their approach, on the one hand, executes a generic algorithm subroutine, and on the other hand, solves a set-partitioning model to recombine the routes encountered during the search process. In the genetic algorithm, an LNS is embedded to act as a mutation operator. At the end of each iteration, the local search procedure is performed to further improve the solutions generated by the genetic algorithm and set-partitioning model.

Macrina et al. (2019a) presented a new variant of the GVRPTW that optimizes a mixed vehicle fleet composed of EVs and CVs. The EVs can be partially recharged at any recharging station. The objective function of this problem is the sum of four terms, which are the cost of energy recharged by all EVs, fixed costs of the EVs, variable traveling costs of the EVs, and variable traveling costs of the CVs. For this problem, the authors proposed an iterated local search heuristic that mainly consists of a perturbation procedure and a local search procedure. Through experiments, they showed how the time windows and partial recharging policy affect the solution quality. Subsequently, Macrina et al. (2019b) extended the problem by incorporating: 1) a comprehensive energy consumption function that considers speed, acceleration, deceleration, loaded cargoes, and road gradients; 2) the effects of the acceleration and braking phases; and 3) the realistic features related to the battery lifespan. Macrina et al. (2019a) only used a standard LNS heuristic to solve their problem and did not reveal the details of their approach.

## 6 Electric location routing problem

Before routing EVs, we need to know the locations of recharging (or battery swapping) stations. In most of the literature, the locations of recharging stations are known in advance. However, for some situations, we need to simultaneously decide on the locations of recharging stations and schedules of EVs, which leads to the research on the ELRP. The ELRP has a single depot, a set of customers with given demands, a set of candidate locations for building recharging stations, and a fleet of identical EVs to be dispatched to delivery cargoes from the depot to the customers. Each customer can be serviced by exactly one vehicle. Each EV must start from and end at the depot, and the amount of loaded cargoes cannot exceed the vehicle capacity. A fixed construction cost exists for building a recharging station, and the shipping cost is proportional to the traveling distance of the EVs. The objective of this problem is to determine the locations of the recharging stations and the routes of all EVs while minimizing the total cost and satisfying all customer demands. Table 4 lists the recent studies on the ELRP and its variants.

Yang and Sun (2015) presented an ELRP that aims to simultaneously determine the locations of the battery swapping stations and the routing plan of a fleet of EVs. The authors first provided a basic mathematical model with an assumption that each vehicle can only visit each swapping station or each customer at most once (see Fig. 9(a)). Subsequently, they removed this assumption and gave an extended mathematical model in which an EV can visit any swapping station multiple times (see Fig. 9(b)). To solve this problem, the authors proposed a two-phase heuristic that combines tabu search algorithm and modified Clarke–Wright saving algorithm (henceforth referred to as TS–MCWS) and a four-phase heuristic called SIGALNS. In the TS–MCWS, the tabu search algorithm is used to fix the locations of the swapping stations, and then the modified Clarke–Wright saving method is executed to decide the vehicle routes based on the given locations of the swapping stations. In the first stage of the SIGALNS, a modified sweep algorithm is invoked to generate an initial solution. In the second phase, a subset of candidate-swapping stations is selected and allocated to different routes using an iterated greedy algorithm. An ALNS heuristic is performed to route the vehicles in the third phase and solutions are further improved by a split procedure in the last phase. Hof et al. (2017) showed how to solve the ELRP by adapting the adaptive VNS (AVNS) heuristic for the VRP with intermediate stops (Schneider et al., 2015). They conducted experiments using the benchmark instances generated by Yang and Sun (2015). The AVNS heuristic significantly improved the previously best-known solutions for the majority of

**Table 4** ELRP and its variants

| Reference | Problem name | Features | Approaches |
|---|---|---|---|
| Yang and Sun (2015) | Battery swap station location-routing problem with capacitated electric vehicles | Locations of battery-swapping stations and EV routes | Two-phase heuristic that combines tabu search algorithm and modified Clarke–Wright saving algorithm and SIGALNS |
| Hof et al. (2017) | Battery swap station location-routing problem with capacitated electric vehicles | Locations of battery-swapping stations and EV routes | Adaptive variable neighborhood search heuristic |
| Schiffer and Walther (2017) | ELRP with time windows and partial recharging | Locations of battery-swapping stations, EV routes, time windows, and partial recharging | MIP model without tailored methods |
| Zhang et al. (2019) | Location-routing problem in EV transportation with stochastic demands | Locations of battery-swapping stations, EV routes, and stochastic demands | Hybrid heuristic composed of a binary PSO algorithm and a variable neighborhood search heuristic |
| Koç et al. (2019) | EVRP with shared charging stations | Locations of battery-swapping stations, EV routes, multiple depots, and investment in charging stations | Multi-start adaptive large neighborhood search heuristic |

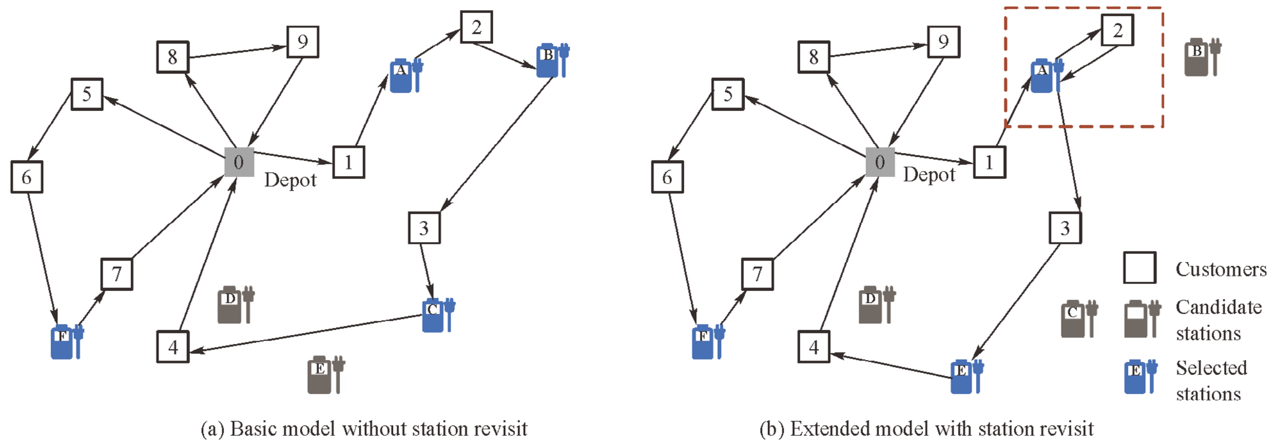

**Fig. 9** Comparison between allowing and not allowing revisit in ELRP.

instances within reduced computation times, compared with the approaches used by Yang and Sun (2015). Moreover, they observed that the AVNS heuristic is robust based on the average solution quality, and is able to considerably reduce the number of swapping stations constructed, compared with the results in the literature.

Schiffer and Walther (2017) focused on the ELRP with time windows and partial recharging policy, which is used to support strategic decisions of transportation companies. The authors formulated their problem into an MIP model and presented four objective functions, each corresponding to one of four scenarios. Thereafter, they derived a strengthened MIP model to reduce the computation time. The authors did not provide any tailored solution procedure for their problem and only directly solved their models through a general MIP solver using small instances with up to 15 customers. By experiments, they showed the advantages of their strengthened model, the benefits brought about by the partial recharging policy, and the effect of different objective functions. Zhang et al. (2019) addressed an ELRP with stochastic demands, with the aim to seek a min-cost solution that outputs the optimal locations of the battery-swapping stations and optimal priori routing plan. They adapted the classical recourse policy and preventive restocking policy to consider the influences of battery capacity. The EV needs to select an optimal swapping station sequence based on its current state of charge. In this selection process, the EV must face two conflicting objectives, namely, minimizing the traveling distance and maintaining higher battery level. The authors tried to identify a Pareto optimal set to speed up the selection of swapping stations. They implemented a hybrid heuristic composed of a binary particle swarm optimization (PSO) algorithm and a VNS heuristic to solve their problem. In the experiments, the authors demonstrated the performance of their proposed approach by comparing it with five other heuristics. Koç et al. (2019) presented a multi-depot ELRP, where each company runs a distribution system consisting of a fleet of identical EVs, a depot, and a preassigned set of customers. However, these companies jointly invest in installing and operating recharging stations. That is, the recharging stations are shared by these companies. This problem aims to determine the locations of the recharging stations and schedule EVs for each company. The authors implemented a multi-start ALNS heuristic to solve their problem and evaluated their method using self-generated test instances.

## 7 Other variants

In this section, we review the literature of the EVRP variants that do not appear in the aforementioned context. All articles except two were published in 2019 and thus, the number of articles about each type of variant is limited.

### 7.1 Hybrid electric vehicle routing problem

Mancini (2017) introduced the HEVRP and assumed that the vehicle shifts to fuel propulsion mode only immediately after the power in the vehicle battery is depleted. The authors formulated this problem into an MIP model and then proposed a matheuristic that combines a standard ALNS heuristic with a simplified model. The matheuristic starts from a feasible solution. Two routes are destroyed at each iteration. Then, a simplified model is solved, which only considers two vehicles and the customers in the destroyed two routes to generate two new routes. The aforementioned two steps are iteratively executed until a termination criterion is reached. Yu et al. (2017) developed two versions of simulated annealing algorithm with a restart strategy to solve this problem. These two versions employ the Boltzmann and Cauchy functions to determine the acceptance probability of a worse solution, respectively. We refer the reader to Dascioglu and Tuzkaya (2019) for a review of the papers regarding the HEVRP published in or before 2017. In the following, we survey two most recently published papers.

Li et al. (2018) proposed a multi-objective EVRP that uses plug-in and wireless charging systems. They formulated the problem into an MIP model and solved it by CPLEX efficiently. Zhen et al. (2020) introduced a variant of the HEVRP in which vehicles can run on four types of modes. The hybrid electric vehicles (HEVs) can run as long as they have battery power or gasoline. However, in this work, the impact of the vehicle load on the electricity or gasoline consumption is neglected. This problem needs to decide which gas stations or recharging stations will be visited in the routes of the HEVs. Prices of gasoline and electricity are given, and the amounts of gasoline and electricity consumed on each edge are also known beforehand. This problem has an implicit assumption that each edge can only select exactly one mode. The authors developed an improved PSO algorithm to solve their problem, which is a mixture of the PSO and VNS procedures and uses a label procedure to assign a mode to each edge.

### 7.2 Electric dial-a-ride problem

If the dial-a-ride problem (DARP) uses EVs and allows EVs to be recharged during the trip, then we can obtain the EDARP. The DARP consists of designing vehicle routes for a number of requests, each characterized by a pickup point (origin), a delivery point (destination), and a certain quantity of demand. For all requests, a maximal ride time exists, i.e., the difference of the pickup and delivery times must be within a given limit. For comprehensive reviews on the DARP, we refer the reader to Cordeau and Laporte (2007), Molenbruch et al. (2017), and Ho et al. (2018).

Masmoudi et al. (2018) introduced the EDARP, which arises specifically in healthcare services related to non-emergency transportation of patients. Different patients need to be transported between their homes and the clinics (or hospitals). In this problem, multiple types of EVs are considered and each EV can provide several types of resources, each with a certain capacity. The EDARP aims to plan a set of routes to fulfill all requests while minimizing the total routing cost. A feasible solution to the EDARP must satisfy the following conditions: 1) the pickup vertex must be visited before its corresponding delivery vertex, 2) the constraints of the resource capacities must be respected, 3) the service of each vertex must be started within its time window, 4) the limitation on the ride time for each patience cannot be violated, and 5) each request must be fulfilled by exactly one EV. To solve the problem, the authors proposed three versions of the evolutionary VNS heuristic in which the VNS heuristic is integrated into the framework of the genetic algorithm. In addition, the techniques of the shuffled frog-leaping algorithm (Eusuff et al., 2006) and the bees algorithm are also used in their approaches. Shi et al. (2018) also studied the EDARP in the context of ride-sharing service and formulated the problem into an MIP model.

Bongiovanni et al. (2019) extended the work of Masmoudi et al. (2018) by considering multiple starting and ending depots. Each EV can return to one of the candidate ending depots rather than return to its starting depot. The recharging stations can only be accessed by empty EVs and can provide partial recharging service. The authors formulated their problem as 3-index and 2-index MIP models, and then devised a branch-and-cut algorithm with new valid inequalities derived from the problem structure. By experiments, they found that solving the 2-index model can yield better results compared with the 3-index model. The largest instances optimally solved by their approaches contain up to 5 EVs and 40 requests. Al-Kanj et al. (2020) addressed a comprehensive ride-hailing system that dispatches a centrally managed fleet of autonomous EVs. They used approximate dynamic programming algorithm to determine trip assignment and recharging operations.

### 7.3 Electric two-echelon vehicle routing problem

Two-echelon distribution systems are common in the industry of logistics, necessitating the investigation of the two-echelon VRP (2EVRP). In the 2EVRP, the cargoes are first transported from depot to intermediate points (called satellites) by large vehicles and then distributed from satellites to customers by small vehicles. At the satellites, the cargoes need to be transferred from large vehicles to small vehicles. Therefore, we need to simultaneously handle two VRPs, namely, routing large vehicles from a depot to a set of satellites (i.e., first echelon vehicle routing) and routing small vehicles from a satellite to a set of customers (i.e., second echelon vehicle routing). For more information with regard to 2EVRP, we refer the reader to Perboli et al. (2011), Baldacci et al. (2013), and Jepsen et al. (2013). When this problem uses EVs to transport cargoes, we can obtain an extended variant called E2EVRP. Figure 10 provides an example of the E2EVRP transportation network.

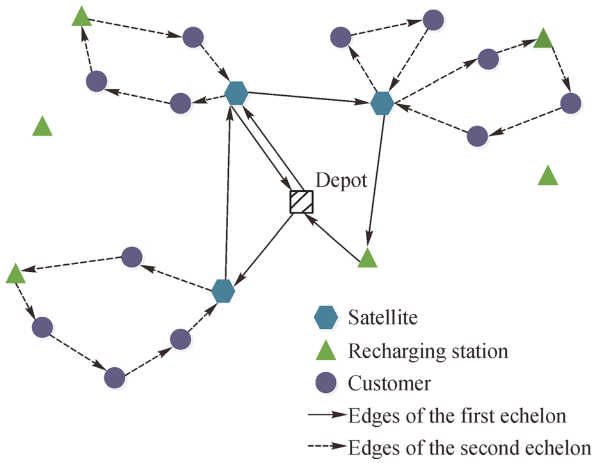

**Fig. 10** Example of E2EVRP transportation network.

Breunig et al. (2019) proposed an E2EVRP that only uses EVs in the second echelon distribution stage. They developed an exact method to solve this problem to optimality based on the method in Baldacci et al. (2013) for the 2EVRP. From their experimental analysis, the authors found that their exact algorithm can only optimally solve small- and medium-sized instances. For the large instances, they designed an LNS heuristic to produce high-quality upper bounds. Jie et al. (2019) studied an E2EVRP that uses EVs in both echelons. They solved their problem using a hybrid heuristic called CG–ALNS, which is composed of a column generation (CG) procedure and an ALNS heuristic. The routing problem for the first echelon, which is handled by the CG procedure, is a split-delivery EVRP. The routing problem that is solved by the ALNS heuristic in the second echelon can be regarded as a multi-depot EVRP by assuming satellites to be depots. The authors implicitly assumed that the customer demands are integral, which can be revealed from the description of the column generation procedure in their study.

### 7.4 Electric pickup and delivery problem with time windows

The pickup and delivery problem with time window (PDPTW) has been widely studied by many researchers. In the PDPTW, $n$ transportation requests need to be fulfilled. A transportation request comprises an origin point and a destination point. A given amount of commodity is picked up from the origin point and then delivered to its corresponding destination point. Each operation has a service time and must be started within a given time window. A fleet of identical vehicles with a known capacity is dispatched to fulfill these requests. All vehicles must start from and end at the depot. If we replace CVs with EVs, then the PDPTW becomes EPDPTW. Goeke (2019) introduced a compact formulation of the EPDPTW with the partial recharging policy and developed the first solution approach, which is called granular tabu search (GTS) algorithm. The idea behind the GTS algorithm is not new and has been applied to several other VRPs, such as Toth and Vigo (2003) and Escobar et al. (2014). In their algorithm, they effectively handled the partial recharges when time window constraints were allowed to be violated. No benchmark approach can be found in the literature. Therefore, the author applied the algorithm to solve the PDPTW and compared their results with those generated by five best PDPTW algorithms available in the literature. The comparison results showed that the GTS algorithm can produce highly competitive solutions.

## 8 Conclusions

We presented a comprehensive literature review on the routing problems concerning EVs. All the surveyed papers are extended from their corresponding vehicle routing counterparts. Two types of recharging policies, namely, full and partial recharging policy, can be adopted by the EVRPs. Thus, each problem can have two versions. The partial recharging policy is more flexible while increasing the complexity of the problem and the difficulty of the solution approach. Many studies have demonstrated the benefits resulted from the adoption of the partial charging policy. In addition, numerous papers have incorporated the battery-charging functions and energy consumption functions into their models and made their problem closer to the real practice. In terms of the solution approaches, most of the works inherited the methods for VRP and made some changes based on the properties of EVs. Further efforts have been exerted to decide the locations of the recharging (or battery-swapping) stations and when to recharge the EVs.

After reviewing these papers, we found several obvious research gaps, which are described as follows. No existing paper reports real industrial cases that truly applied the models and algorithms of the EVRPs. Therefore, we have no clue regarding the companies that applied the relative methods and cost reduction caused by adopting the EVs. The scale of the instances handled in the literature is far smaller than the scale of real instances, which usually involves hundreds of EVs and thousands of customers. The methods suitable for the small instances are likely inapplicable to the real and large instances. We cannot find any existing study focused on designing algorithms to solve large-scale instances. Some researchers have devised reinforcement learning algorithms to solve the traditional VRP while such methodologies have yet to deal with EVRPs. Car-sharing and online car-hailing businesses, such as Uber, have been operating in many countries and are using EVs prevalently. No research problem has been introduced and studied in such backgrounds. Range anxiety is the fear that a vehicle has insufficient range to reach its destination. Uncertain environments and tasks considerably aggravate range anxiety. Few studies have paid attention to the solution approaches for the EVRPs under uncertain environments and random customer requests.

The literature on the EVRP is not yet extensive and therefore, more researchers can contribute to this area. Four promising avenues are available for further research. First, in the models, we can consider mobile recharging stations and thus make the transportation more flexible. Second, almost all VRPs can be upgraded by replacing CVs with EVs. Therefore, we can attempt to extend other VRP variants to their electric version. Third, we observed from this survey that the research on exact algorithms is scarce, and more researchers are needed to design the approaches for the optimal solution of the problem. Finally, with the arrival of driverless vehicles and the era of 5G, additional problems, models, and algorithms related to the routing of EVs can be proposed and investigated.

**Acknowledgements** This study was written in early 2020, when the first author was infected with COVID-19. Fortunately, he received timely free treatment and recovered in time to submit this review paper. We are grateful to the Chinese government and all the people who supported the first author.

**Open Access** This article is licensed under a Creative Commons Attribution 4.0 International License, which permits use, sharing, adaptation, distribution and reproduction in any medium or format, as long as you give appropriate credit to the original author(s) and the source, provide a link to the Creative Commons licence, and indicate if changes were made.

The images or other third party material in this article are included in the article's Creative Commons licence, unless indicated otherwise in a credit line to the material. If material is not included in the article's Creative Commons licence and your intended use is not permitted by statutory regulation or exceeds the permitted use, you will need to obtain permission directly from the copyright holder. To view a copy of this licence, visit http://creativecommons.org/licenses/by/4.0/.
